# Deep learning-based prediction of intra-cardiac blood flow in long-axis cine magnetic resonance imaging

**DOI:** 10.1007/s10554-023-02804-2

**Published:** 2023-02-10

**Authors:** Xiaowu Sun, Li-Hsin Cheng, Sven Plein, Pankaj Garg, Mehdi H. Moghari, Rob J. van der Geest

**Affiliations:** 1 Division of Image Processing, Department of Radiology, Leiden University Medical Center, Leiden, The Netherlands; 2 Leeds Institute of Cardiovascular and Metabolic Medicine, University of Leeds, Leeds, UK; 3 Norwich Medical School, University of East Anglia, Norwich, UK; 4 Norfolk and Norwich University Hospital Foundation Trust, Norwich, UK; 5 Department of Radiology, Children’s Hospital Colorado, and School of Medicine, The University of Colorado, Boulder, CO USA

**Keywords:** Blood flow pattern, 4D flow MRI, Deep learning, Cardiac MRI, Velocity

## Abstract

**Purpose:** We aimed to design and evaluate a deep learning-based method to automatically predict the time-varying in-plane blood flow velocity within the cardiac cavities in long-axis cine MRI, validated against 4D flow. **Methods:** A convolutional neural network (CNN) was implemented, taking cine MRI as the input and the in-plane velocity derived from the 4D flow acquisition as the ground truth. The method was evaluated using velocity vector end-point error (EPE) and angle error. Additionally, the E/A ratio and diastolic function classification derived from the predicted velocities were compared to those derived from 4D flow. **Results:** For intra-cardiac pixels with a velocity > 5 cm/s, our method achieved an EPE of 8.65 cm/s and angle error of 41.27°. For pixels with a velocity > 25 cm/s, the angle error significantly degraded to 19.26°. Although the averaged blood flow velocity prediction was under-estimated by 26.69%, the high correlation (PCC = 0.95) of global time-varying velocity and the visual evaluation demonstrate a good agreement between our prediction and 4D flow data. The E/A ratio was derived with minimal bias, but with considerable mean absolute error of 0.39 and wide limits of agreement. The diastolic function classification showed a high accuracy of 86.9%. **Conclusion:** Using a deep learning-based algorithm, intra-cardiac blood flow velocities can be predicted from long-axis cine MRI with high correlation with 4D flow derived velocities. Visualization of the derived velocities provides adjunct functional information and may potentially be used to derive the E/A ratio from conventional CMR exams.

**Supplementary Information:**

The online version contains supplementary material available at 10.1007/s10554-023-02804-2.

## Introduction

Assessment of cardiac function using cardiac magnetic resonance imaging (CMR) is typically based on cine MR imaging. Four-dimensional (4D) flow MRI enables time-resolved three-dimensional visualization of intra-cardiac blood flow to gain a better understanding of the patient’s cardiac condition [1, 2]. Cardiac dysfunction is strongly associated with abnormal patterns of blood flow within the cardiac chambers. Therefore, visualization and quantification of intra-cardiac blood flow may provide relevant diagnostic information. However, 4D flow MRI is usually not performed in routine clinical protocols as it requires additional scan time and post-processing. During post-processing typically registration is required of the 4D flow acquisition with the acquired long-axis and short-axis cine views, which may be hampered by variations in respiratory condition and heart rate [3, 4]. Interestingly, in standard long-axis cine MR views, the intensity fluctuations within the cardiac cavities provide a visual clue about the global blood flow pattern. While the signal intensity variations are dependent on various factors such as saturation effects and spin dephasing due to magnetic field inhomogeneity or complex flow [5, 6], we speculate that time-varying flow velocity information can be derived from those intensity variations.

There have been many attempts in using balanced steady-state free precession (SSFP) MR imaging for measuring blood velocity by modifying the SSFP sequence. Markl et al. measured through-plane flow using a SSFP sequence by inverting the slice encode gradient between two consecutive acquisitions [7]. The through-plane velocity was then calculated by subtracting the resulting phase images. Nielsen et al. augmented the slice encode gradient in the SSFP sequence for measuring blood velocity in a readout direction [8]. They used the resultant phase information without a reference for measuring the blood velocity in the readout direction. In recent years, convolutional neural networks (CNN) have been introduced to extract cardiac motion information, which could be interpreted as an ensemble of relatively small, periodical variations of the shape and position of heart structures during a cardiac cycle [9–11]. However, the potential application of CNN’s for velocity field prediction has not been explored yet.

Accordingly, in this work we propose a deep learning-based method to track the blood flow displacement within consecutive cardiac frames from long-axis cine MR imaging. As ground truth, we used the velocity field derived from registered 4D flow MRI. Once the blood flow is tracked and the displacement vectors in X and Y directions are measured, pixel wise blood velocity in each direction can be derived by dividing its displacements to the temporal resolution of each frame. To the best of our knowledge, we are the first to employ deep learning and 4D flow MRI for automated cardiac blood flow prediction. Additionally, in clinical routine, diastolic function is usually evaluated using Doppler echocardiography. Although, several studies demonstrated the usefulness of CMR in deriving conventional diastolic parameters, those methods rely on additional scan time and extra post-processing, such as the manual localization of regions of interest (ROI), which is time-consuming [12–14]. In our work the E/A ratio is automatically derived from the predicted blood flow and used to classify diastolic function as a potential clinical application.

## Methods

### Dataset

The study cohort included 78 post-myocardial infarction (MI) patients and 34 healthy subjects who underwent cardiac MRI on a 1.5T MR system (Philips Healthcare). The study was approved by the local medical ethical committee and all participant in the study provided written information consent. The MR imaging protocol included conventional SSFP cine in 4-chamber (4CH) view and a short-axis cine stack. In addition, whole-heart 4D flow MRI was performed for 3D blood flow velocity assessment in the four cardiac chambers. Both cine MRI and 4D flow MRI were reconstructed into 30 phases covering a complete cardiac cycle. MR imaging parameters of the acquisitions are listed in Table 1. More details about the MR acquisition protocol have been reported in earlier work [15, 16].

**Table 1:** 4D flow and SSFP data acquisition parameters. VENC: velocity encoding; FOV: field of view; TE: echo time; TR: repetition time; bpm: beats per minute

	4D Flow Data	SSFP
Spatial resolution (mm^3^ )	3 × 3 × 3	0.95–1.25 × 0.95–1.25 × 8
Reconstructed temporal resolution (ms)	20.83–46.73	20.21–48.21
Electrocardiogram gating	retrospective	retrospective
VENC (cm/s)	150	—
FOV (mm^2^ )	300–440 × 300–440	300–440 × 300–440
TE/TR (ms)	3.10–3.75/7.46–13.95	1.5–1.72/3.0-3.44
Flip angle (°)	10	60
Reconstructed heart phases	30	30
Scan time	7–10 min	6–8 s
Heart rate (bpm)	41–94	42–99
Motion correction	None (free breathing)	Breath hold

Mass software (Version V2017-EXP; Leiden University Medical Center, Leiden, the Netherlands) was used to derive LV volumetric parameters from the short-axis cine stack by semi-automated segmentation of the endocardial and epicardial borders. The semi-automatically defined ventricular and atrial contours in the 4CH view were used as a mask and for each pixel within the mask the in-plane component of velocity as derived from the aligned 4D flow acquisition was used as the ground truth. To avoid temporal inconsistency, cine acquisitions were excluded if the heart rate deviated from that of the 4D flow acquisition by more than six beats per minute. Based on this exclusion criterion, 92 cases (2760 2D images) remained for training and testing. Table 2 summarizes the detailed demographics derived from the short-axis cine and 4D flow data.

**Table 2:** Demographics of the study cohort derived from the short-axis cine and 4D flow data. Data is presented as mean ± standard deviation or count. EDV: End-diastolic volume, ESV: End-systolic volume, SV: Stroke volume, EF: Ejection fraction

Characteristic	Subjects(n = 92)
Gender (Male, n)	56
EDV (ml)	179.71 ± 63.93
ESV (ml)	90.14 ± 58.27
SV (ml)	89.58 ± 19.38
EF (%)	53.11 ± 12.27
E/A ratio	1.41 ± 0.54

In-plane spatial alignment was performed between the SSFP cine and reformatted 4D flow images since 4D flow images were acquired during free-breathing while SSFP cine images were acquired during breath-hold. In addition, significant patient motion can occur in between the acquisition of the long-axis cine view and the 4D flow acquisition. Based on the image position information, the in-plane velocity derived from 4D flow was projected on the cine long-axis views. In case a misalignment was observed between the visualized anatomy and the velocity vectors, the cine view images were manually translated in order to optimize the alignment. We further assumed that both 4D flow and SSFP cine images are registered in time since both have the same number of cardiac phases and nearly similar heart rates. Therefore, each cardiac phase of 4D flow is assumed to correspondent to same cardiac phase of SSFP cine. Tri-linear interpolation was used to generate the in-plane velocity components for the 4CH long-axis view.

### Data preprocessing

In this work, we aim to predict the blood flow velocity within the cardiac chambers. To filter out irrelevant velocity information, we applied a binary blood pool mask in the long-axis view to exclude the region outside of the cardiac chambers. The signal intensities of the input cine sequence were normalized based on the histogram of the signal intensities within the masked region. The histogram was constructed by aggregating the blood pool pixels of all cardiac phases, which implies that signal loss information is still preserved and flow-induced artifacts can still be tracked from frame to frame. The normalization can be described as in formula 1, where ${P}_{\text{n}\text{o}\text{r}\text{m}-\text{i}}$, the normalized value of the pixel-*i* is derived from ${P}_{I}$ the signal intensity of pixel-*i*, ${P}_{5th}$ and ${P}_{95th}$ represent the 5th and 95th percentile value of the intensity histogram.

1 $${\varvec{P}}_{\varvec{n}\varvec{o}\varvec{r}\varvec{m}-\varvec{i}}=\frac{{\varvec{P}}_{\varvec{I}}-{\varvec{P}}_{5\varvec{t}\varvec{h}}}{{\varvec{P}}_{95\varvec{t}\varvec{h}}-{\varvec{P}}_{5\varvec{t}\varvec{h}}}$$

The intensity fluctuations in the cine MR sequence are used to predict the displacement of a pixel, i.e. a blood sample, from frame to frame. However, the 4D flow acquisition provides each pixel’s velocity instead of displacement. Therefore, the pixel velocities derived from the 4D flow acquisition are converted into the pixel displacements using formula 2,

2 $$\mathbf{D}=(\frac{\varDelta t{v}_{x}}{p{s}_{x}},\frac{\varDelta t{v}_{y}}{p{s}_{y}})$$

in which $\mathbf{V}=({v}_{x},{v}_{y})$ stands for velocity of each pixel in frame t, ${v}_{x},{v}_{y}$are the velocities projected on the long-axis image, ∆t is the time interval between image frame t and t + 1 and $\mathbf{P}\mathbf{S}=({ps}_{x},{ps}_{y})$ is the pixel spacing. After this preprocessing, the displacement **D** (in pixel units) from frame t to frame t + 1 is regarded as the ground truth for model training.

### Network structure

**Fig. 1:**
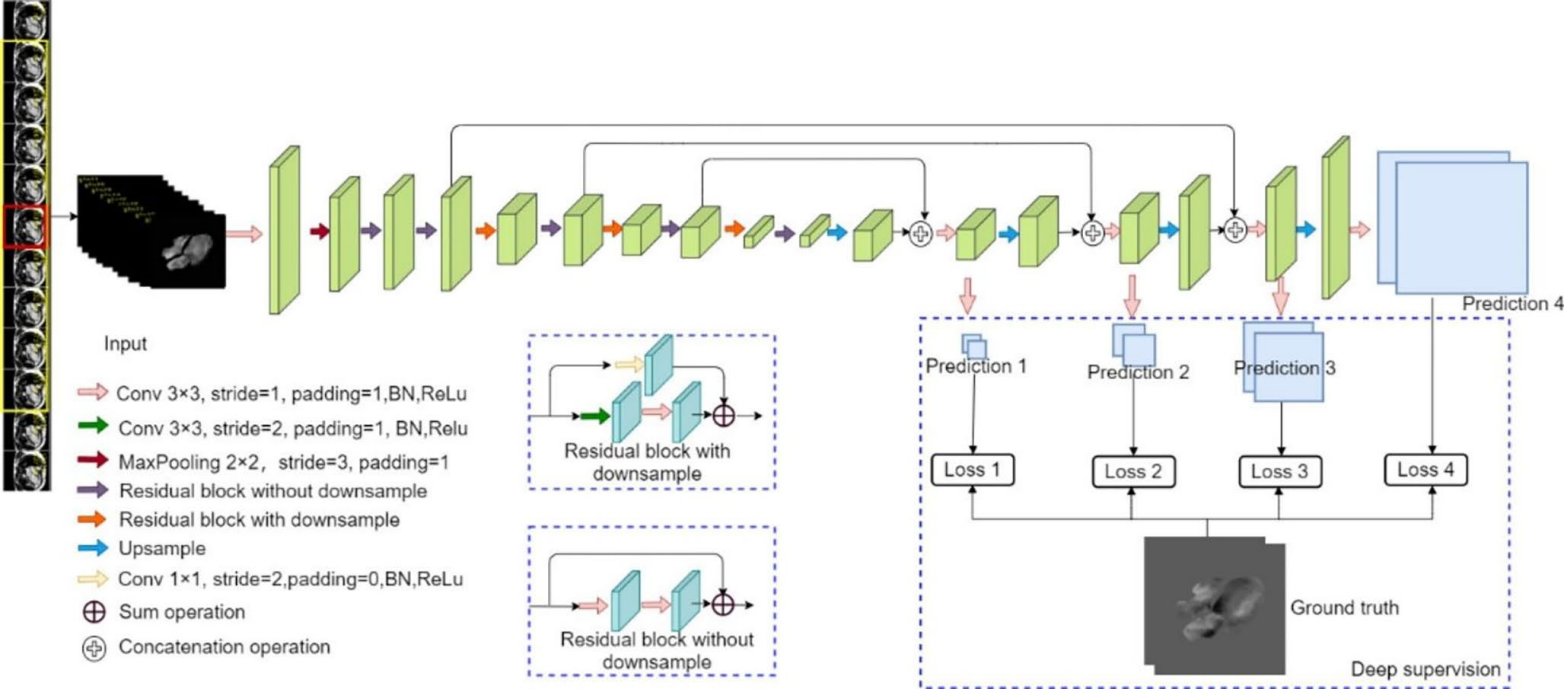
Architecture of the proposed network. The input of the network is a sub-sequence of 2D cine MR images including the target image (in red box) and its 8 temporal neighboring frames (in yellow box). Prediction 1,2,3,4 are four outputs with two dimensions on X and Y directions, respectively, which are used to compute the deep supervision loss. Prediction 4 is the final output of the network and is the one evaluated during the test. Only the pixels within the blood pool region were used to be evaluated by the network.

The displacement information and moving direction of a pixel, or group of pixels, can only be extracted using the current and its neighboring frames. To predict the in-plane components of blood flow velocity, we consider a sequence of cine MR images containing a central image and its 8 temporal neighboring phases as the input and the displacements in X and Y direction derived from the 4D flow sequence as the ground truth to train an end-to-end network. The proposed CNN architecture is illustrated in Fig. 1.

The implemented network is a variant of U-Net [17] and ResNet [18] containing a contracting path and an expanding path. In the contracting path, to provide dense per-pixel predictions, one pooling operation and three strided convolutions with a 1 × 1 kernel size are applied for the down-sampling. The conventional convolution layers in the contracting path of U-Net are replaced with residual convolution modules to extend and deepen the network. In the expanding path, we reserved the concatenation-based skip connections to integrate the local features and the global information.

Deep supervision [19] is employed to overcome the problem of vanishing gradients in a deep CNN architecture. As shown in Fig. 1, three auxiliary prediction layers are inserted before the up-sampling operation, each prediction is resampled into the original image size using nearest neighbor interpolation. The end point error (EPE), being the Euclidean distance between two displacement vectors averaged over all pixels within the cardiac cavities, is used as loss function. Given ${D}_{x,g},{D}_{y,g},{D}_{x,p},{D}_{y,p}$ representing the displacement values of ground truth and prediction in X and Y directions, ${\mathbf{D}}_{i,g}=({D}_{x,g},{D}_{y,g})$ and ${\mathbf{D}}_{i,p}=({D}_{x,p},{D}_{y,p})$ denoting the displacement vectors for ground truth and prediction of *i*^th^ pixel within the blood pool, then the EPE is defined according to formula (3) where *M* indicates the number of pixels within the blood pool.

3 $$\text{E}\text{P}\text{E}=\frac{1}{M}{\sum }_{i=0}^{M}||{\mathbf{D}}_{i,p}-{\mathbf{D}}_{i,g}||=\frac{1}{M}{\sum }_{i=0}^{M}\sqrt{{({D}_{x,p}-{D}_{x,g})}^{2}+{({D}_{y,p}-{D}_{y,g})}^{2}}$$

The EPE loss is the sum of length of the displacement vector difference to compute the magnitude and angle error between prediction and ground truth for all pixels within the blood pool. The total loss is defined as:

4 $$\text{L}\text{o}\text{s}\text{s}=\text{E}\text{P}\text{E} \left(\text{G},\text{O}\right)+\sum _{c}{w}_{c}{EPE}_{c}(G,{P}_{c})$$

where G is the displacement generated from the 4D flow data, $\text{O}$ is the final output from the network, ${P}_{c}$ is the prediction of the c^th^ auxiliary prediction layer and ${w}_{c}$ is the loss weight of each auxiliary prediction.

To improve the performance and the generalization of the model, five-fold cross-validation was applied. The output of CNN was divided by the temporal resolution to convert to velocity to compute the evaluation metrics.

### Evaluation metrics

#### Visual evaluation

To visually assess the intra-cardiac blood flow patterns derived from either the CNN prediction and 4D flow, the in-plane velocity was displayed in movie mode as vector overlay projected on the cine MR images. The length and color of the displayed vectors were scaled according to the velocity magnitude.

#### Quantitative evaluation metrics

The performance of the proposed method was evaluated using EPE and angle error.

To quantitatively assess the performance of the blood flow prediction, both the magnitude and angle error are required to be measured. Therefore, EPE described in formula 3 and trigonometric function are employed to compute the magnitude and angle error, respectively. Here, the EPE was computed using the velocity vectors instead of the displacement vectors. The angle error $\theta$, between the ground truth ${\mathbf{V}}_{i,g}$ and prediction ${\mathbf{V}}_{i,p}$ of the *i*^th^ pixel within the blood pool, is defined as,

5 $${\uptheta }=\frac{1}{M}\sum _{i=0}^{M}\text{a}\text{r}\text{c}\text{c}\text{o}\text{s}\left(\frac{{\mathbf{V}}_{i,p}\bullet {\mathbf{V}}_{i,g}}{||{\mathbf{V}}_{i,p}|| ||{\mathbf{V}}_{i,g}||}\right)$$

where *i* represents the *i*^th^ pixel and *M* indicates the total number of pixels within in the blood pool,$||\bullet ||$ is the length of a vector and *arccos* means the inverse trigonometric function of cosine. The angle error ranges between 0° and 180°, with 0° denoting two vectors in the same direction and 180° denoting two vectors in opposite direction.

#### Clinical parameters

A commonly clinically used flow-related parameter is the E/A ratio. The E/A ratio can be used to classify diastolic function as either normal or abnormal using the cutoff values for E/A ratio as commonly used in cardiac ultrasound. In our work, a region of interest was first defined by three points, being two end points of the defined LV endocardial contour, which correspond to the valve hinge points, and a third point in the center of LV cavity. A b-spline curve was fitted through the three points, resulting in a region just below the mitral valve plane. The E and A velocities were found by searching for the pixel with maximum (in-plane) velocity within the region to derive the E/A ratio.

#### Statistical analysis

Results are expressed as mean ± standard deviation (SD). Pearson correlation coefficient (PCC) was used to evaluate the correlation between our prediction and the 4D flow data for the velocity values during a complete cardiac cycle. In addition, Bland-Altman analysis was used to analyze the mean differences (Bias) and limits of agreement (LOA, 1.96 × SD) of the E/A ratio derived from either the deep learning method or 4D flow data. Paired t-test was performed to test the statistical significance of the differences between paired E/A ratio measurements, P < 0.05 indicates a significant difference. PCC was also used to measure the correlation of E/A ratio derived from 4D flow data and our approach.

## Results

We first introduced 9 neighboring cine MR phases in the input (more results using different number of inputs can be found in the Supplementary file), then we reported the predicted results using the defined metrics. At last, the E/A ratio results were reported.

### Visual comparison

**Fig. 2:**
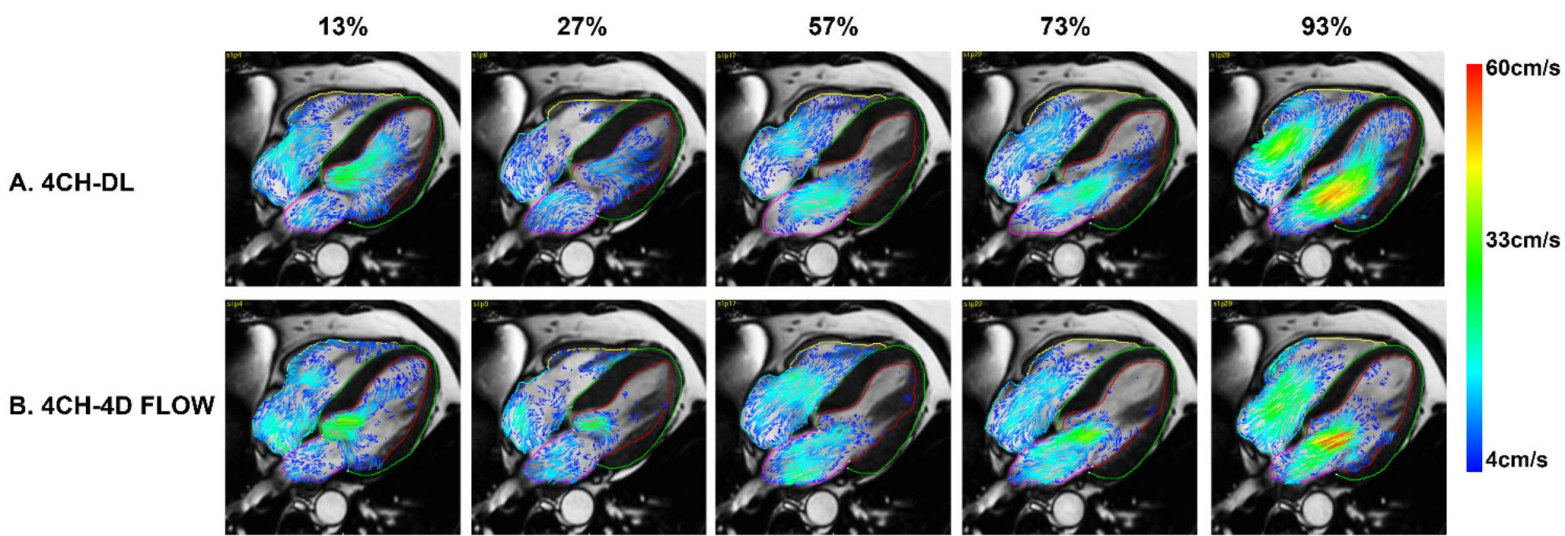
Five out of 30 frames of blood flow pattern generated from deep learning-based method and 4D flow. **(A)**: Blood flow pattern in 4CH view using deep learning. (**B)**: Corresponding ground truth from 4D flow data. The five frames are at 13%, 27%, 57%, 73% and 93% of one cardiac cycle.

The predicted and 4D flow derived in-plane blood flow velocity were dynamically visualized as overlay on the original long-axis cine images. The length and colouring of the vectors were used to encode the local blood velocity magnitude. To avoid cluttering of the vectors and to suppress velocity noise the velocity vectors were only generated for image pixels with a velocity > 4 cm/s. Figure 2 shows an example of selected frames of predicted blood flow velocities compared to 4D flow derived velocities in one of the study subjects. Overall a good agreement is seen in the blood velocity pattern within the cardiac cavities both in systole and diastole. In general it was observed that the visual agreement in flow pattern was better in the ventricles than in the atria. Video examples can be found here (https://github.com/xsunn/BloodFlowPrediction).

### Quantitative results

**Fig. 3:**
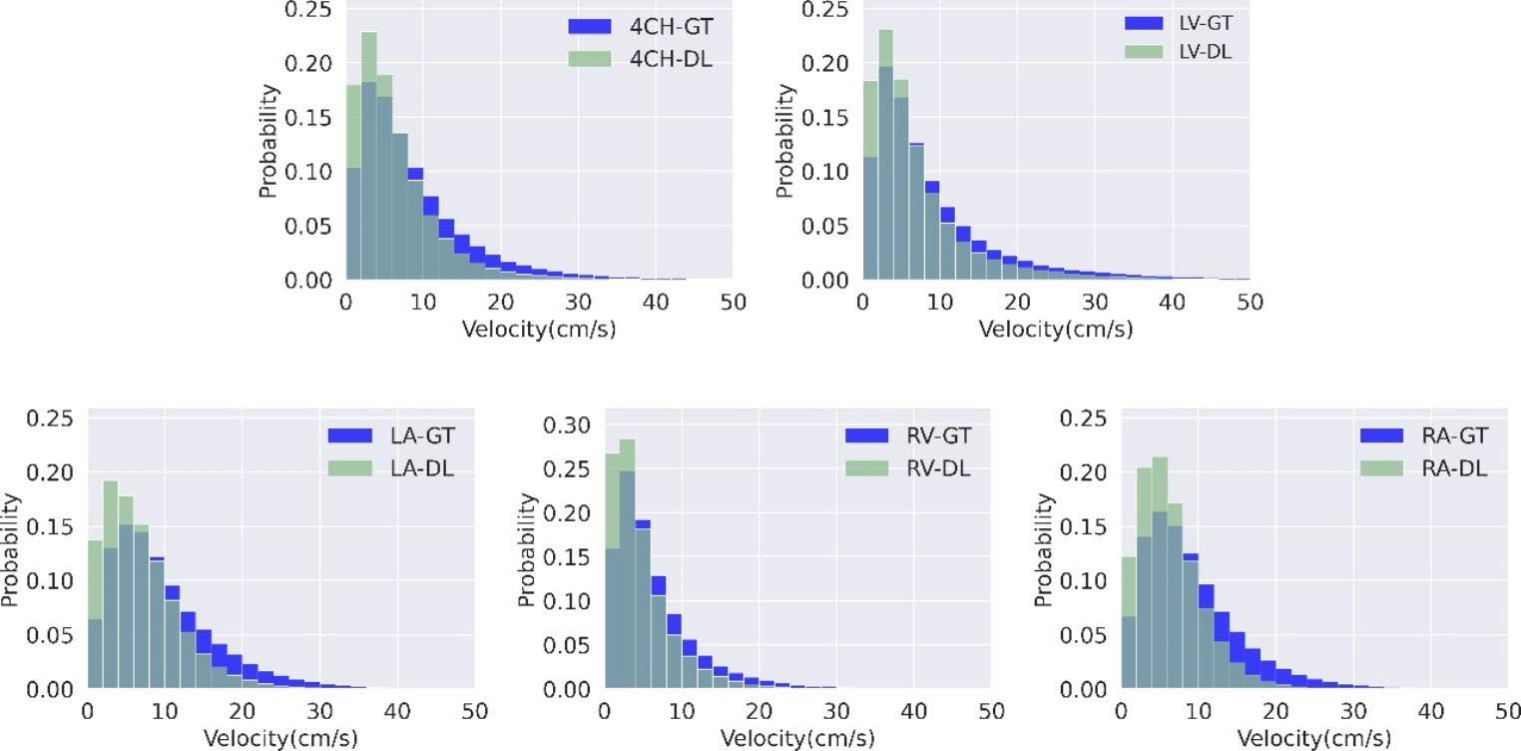
Probability distribution of velocity generated from 4D flow data and prediction. The blue color represents the distribution generated from the 4D flow data, and the light green means the distribution generated from the prediction. The light blue represents the overlap between the prediction and 4D flow data.

Figure 3. shows probability distributions of blood flow velocity in different heart chambers generated from 4D flow data and our prediction. Compared with the ground truth, the predicted velocities were generally lower. 

To quantify the prediction error, those pixels with velocities greater than 5 cm/s were involved in computing the EPE and angle error. The accuracy was computed with 30^th^ percentile as a threshold. All pixels were used to compute the relative error (RE) of velocity between the 4D flow and automated velocity prediction. PCC was used to measure the correlation of the time-varying averaged velocity between the 4D flow data and prediction. The results in different heart chambers are reported in Table 3. The relative error shows that the velocities were under-estimated by 26.69%. The small standard deviation in the relative velocity difference suggests that potentially a constant correction factor may be applied to the predicted velocity to improve the performance. The PCC of velocity within all four chambers was 0.95, indicatings a good correlation in the blood flow pattern between the 4D flow and our prediction.

**Table 3:** Prediction results of different chambers. 4CH indicates the results were computed within all 4 chambers; LV, LA, RV and RA mean the results were based on each single chamber separately. RE: relative error. PCC: Pearson correlation coefficient. The mean ± standard deviation are reported.

	4CH	LV	LA	RV	RA
EPE (cm/s)	8.65 ± 2.69	9.10 ± 2.96	8.45 ± 2.20	7.06 ± 1.54	8.64 ± 2.44
Angle Error (°)	41.27 ± 11.39	37.98 ± 10.94	41.19 ± 12.78	40.99 ± 11.28	47.52 ± 16.90
Velocity-RE(%)	-26.69 ± 4.43	-24.53 ± 4.29	-27.84 ± 6.62	-26.18 ± 8.05	-29.83 ± 4.53
Velocity-PCC	0.95	0.98	0.94	0.93	0.93

Figure 1 in Supplementary shows more details about the performance of our method for different chambers with varying velocity thresholds.

### E/A ratio results

**Fig. 4:**
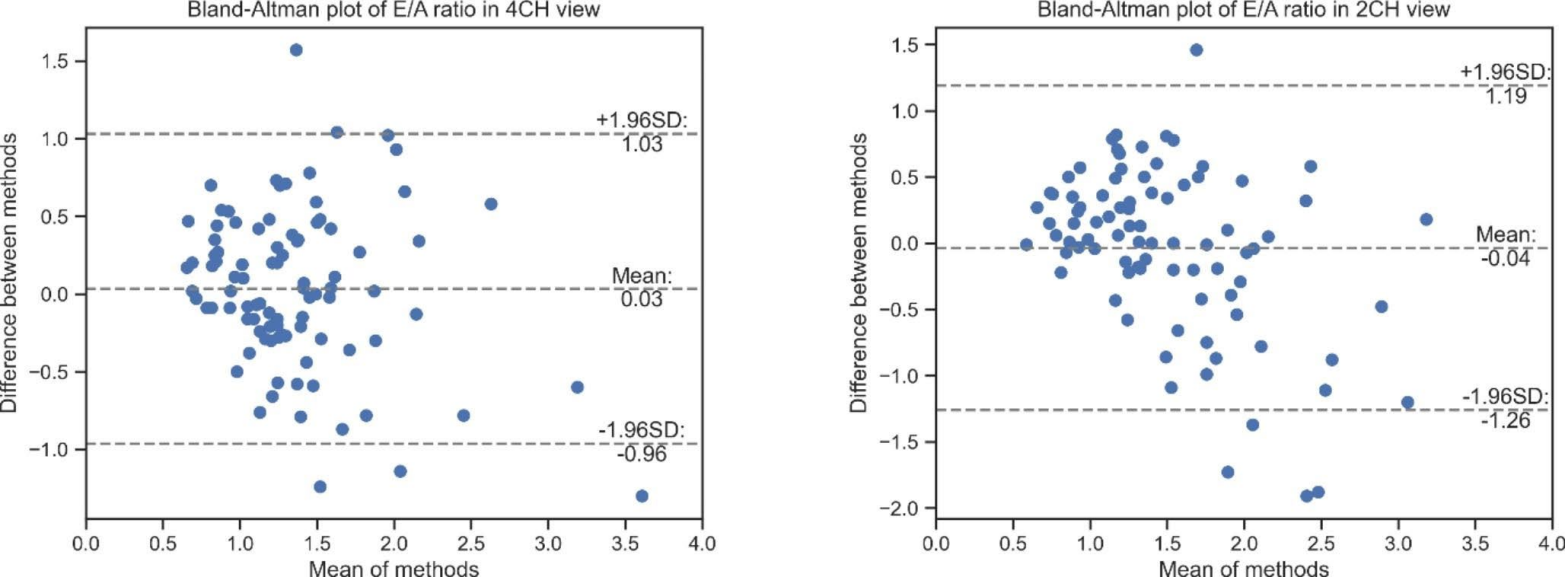
Bland-Altman plots of E/A ratio

The average absolute error in E/A ratio estimation was 0.39 ± 0.32. The Bland-Altman analysis as shown in Fig. 4 reveals a minimal bias with wide limits of agreement (LOA) between our prediction and 4D flow derived E/A ratio and more than 95% of cases are distributed between upper and lower agreement limits.

**Fig. 5:**
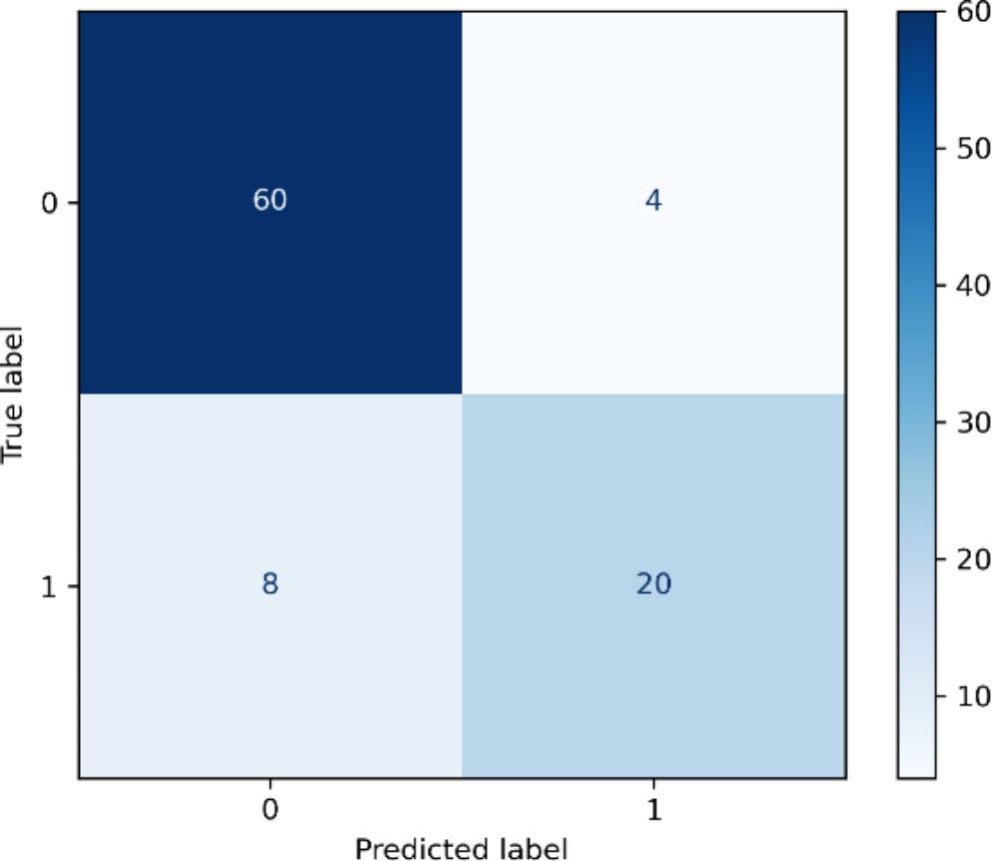
Confusion matrix of diastolic function classification derived from the predicted velocities. Label 0 means normal diastolic function, 1 represents abnormal function.

To investigate the potential clinical applicability of the automated E/A ratio prediction we tested whether the wide LOA effects the classification of diastolic function. Echocardiography is the main imaging modality for assessment of LV diastolic function. It defined 0.75 < E/A ratio < 1.5 as normal diastolic function and E/A ratio varying in the other ranges as abnormal diastolic function [20]. The confusion matrix of the diastolic function classification experiment are summarized in Fig. 5. The diastolic function binary classification accuracy was (60 + 20)/92 = 86.9%. The other three classification metrics including precision, recall and F1-Score, PCC and P values are reported in Table 4. Our method was able to correctly classify 93.75% (60/64) of cases qualified by the 4D flow data as normal diastolic function, and 71.43% (20/28) of the cases with abnormal diastolic function. Due to the wide LOA, the overall PCC of the E/A ratio is 66.71%. The PCC of E/A ratio in the groups with normal and abnormal diastolic function are 39.41% and 75.1%, respectively. But all p values of E/A ratio in both two classes are larger than 0.05. Meanwhile, the p value of 0.795 derived from all 92 subjects also confirmed that the E/A ratio generated from our prediction was not significantly different from the 4D flow data.

**Table 4:** The results of diastolic function classification, PCC and p value of E/A ratio in each class with normal and abnormal diastolic functions

	Recall	Precison	F1-Score	PCC	P value
Normal	93.75%	88.24%	90.91%	39.41%	0.052
Abnormal	71.43%	83.33%	76.92%	75.10%	0.088
Overall	-	-	-	66.71%	0.795

## Discussion

We designed and evaluated a deep learning-based method for the prediction of intra-cardiac blood flow velocity from long-axis cine MRI using 4D flow derived velocities as ground truth. The predicted velocities highly correlated with the 4D flow derived velocities with an overall good visual agreement in time-varying flow pattern. Our work shows a potential clinical application to visualize the blood flow pattern without requiring additional 4D flow data. As the E/A ratio is a well-established clinical parameter used to classify diastolic function, the results demonstrated that the proposed method can be applicable to estimate the E/A ratio without significant bias and to classify diastolic function with a high accuracy. Although the observed underestimation of the predicted velocities and the variability in the derived measurements indicate that further refinement of the deep learning model using a larger patient cohort is warranted, we believe our results demonstrate the potential of the proposed method.

The variation in blood signal intensity in the cine MR images provides information on the direction and magnitude of the blood flow in the cardiac cavities. The observed displacement of the apparent visible structures in the blood pool in subsequent frames reflects the velocity. Therefore, we performed experiments with different number of neighboring phases as input of the network. Using only three phases as input was shown to result in the worst performance. This may be explained by the fact that the small total displacement like just one pixel in three neighboring temporal phases makes the velocity prediction sensitive to the spatial resolution of the cine images. When using more frames as the input the structures can be followed over a larger time window making it less sensitive to the spatial resolution. It was concluded that more than three neighboring phases are required to predict the blood flow pattern and for the final model 9 neighboring phases was used as input.

The high correlation of the time varying velocity averaged over all subjects between our prediction and the 4D flow data, as well as the visual evaluation results, demonstrated a good agreement in the global velocity patterns. However, the velocity values predicted by the proposed model are close to 30% lower than those derived from 4D flow data. In the training data, the low velocities (0–20 cm/s) account for a large proportion which may lead the model to underestimate the velocities in regions of high velocity. In addition, the evaluation results are sensitive to the selected velocity thresholds, because different levels’ velocities are relatively concentrated in certain areas. For example, in the left ventricle, the distribution of the lower velocities are more dispersed and complicated in the apical region. Therefore, it is much harder to predict the irregular movement which leads to a relatively large EPE and angle error. The pixels with higher velocities, such as the blood flow from LA to LV in diastole and from the LV towards the aorta in systole, have a relatively fixed direction of motion. Therefore, the angle error decreased when the velocity thresholds increased. However, since the high velocities only account for a small proportion the model is prone to underestimation of high velocities, resulting in a larger EPE for the pixels with higher velocities.

The E/A ratio derived from the velocities could be assessed without bias since both E- and A-velocity were underestimated similarly. Additionally, the statistical test confirmed that there was no significant difference between 4D flow and CNN derived E/A ratio. However, the Bland-Altman analysis revealed a wide limit of agreement. Despite this, the results of diastolic function classification demonstrated that the variability in E/A ratio had minimal effect on the accuracy of diastolic function classification in our study cohort. Echocardiography allows reliable visualization of blood flow pattern. Vector flow mapping (VFM) in echocardiography uses the mass-conservation principle to estimate the azimuthal component of the flow [21]. VFM has been used in many clinical applications including cardiac function evaluation, valvular diseases diagnosis and congenital heart disease. However, VFM is sensitive to out-of-plane flow and boundary conditions [22]. Additionally, the conventional VFM method is applied only to the left ventricle [23]. Our proposed method can be applied to predict the blood flow in the whole heart from any cine long axis view and does not rely on accurate cardiac boundary segmentation. Since cine MRI acquisitions are routinely acquired in standard CMR exams our method can directly predict the in-plane velocities without requiring additional scan time. The combined visualization of blood flow and myocardial motion provides detailed information about cardiac function and hemodynamics. The clinical value of the developed technique should be evaluated in future clinical studies.

There are several limitations in our study. Velocity underestimation is the main limitation since it is patient dependent and varies across the subjects. The use of appropriate data augmentation techniques to artificially enlarge the available set of training data or introducing a weighted loss function by setting larger weights to higher velocities may result in improved performance of the deep learning model. The ground truth generated by projecting the 4D flow data derived in-plane velocities on the long-axis cine MRI is not a perfect reference, due to heart rate difference and patient movement. The heart rate difference cannot be eliminated completely, even though some cases were excluded to keep the temporal consistency. Registration errors can be corrected for visually by applying in-plane translation of the cine MRI images series. Through-plane misalignment and rotational errors are more difficult to correct for. Additionally, as our method relies on converting predicted pixel displacement to velocity, the limited spatial and temporal resolution of the cine MRI data will have an impact on the velocity magnitude and direction prediction. The 4D flow MRI was acquired during free-breathing while SSFP cine images were acquired during breath-hold, implying a difference in physiological condition of the subject. For regions of low blood flow velocity the noise in the 4D flow data may be non-negligible. Additionally, training and testing the model on a wider range of data from multiple scanner types, centers is also required to gain a further understanding in the potential of the proposed blood flow velocity prediction method. Furthermore it would be valuable to investigate the applicability of our method in patients with valvular regurgitation or stenosis and other patient cohorts with cardiac pathologies associated with abnormal flow patterns, such as patients with dyssynchronous myocardial contraction. Since a full detailed electrocardiographic QRS duration evaluation was not available for the patients in our study, we were unable to perform a patient sub-group analysis.

In conclusion, we proposed a deep learning-based method for automated intra-cardiac blood flow velocity prediction from standard long-axis cine MRI. It was demonstrated that, although the predicted velocity magnitude is underestimated, the global velocity patterns show good correlation with the blood flow patterns derived from 4D flow MRI. The method enables estimation of E/A ratio without significant bias, but with wide limits of agreement. After further improvement of the velocity prediction model the method could potentially be valuable for clinical application.

## Electronic supplementary material

Below is the link to the electronic supplementary material.

Supplementary Material 1
