# Supplementary material for: Deep learning-based prediction of intra-cardiac blood flow in long-axis cine magnetic resonance imaging
Source: Int J Cardiovasc Imaging. 2023 Feb 10;39(5):1045–53. doi: 10.1007/s10554-023-02804-2 (PMC10160163; doi:10.1007/s10554-023-02804-2)
Supplement: Supplementary file 1 — Supplementary Material 1 [file 10554_2023_2804_MOESM1_ESM.docx]

Supplementary

## **S1. Evaluation** **metrics**

As the third error metric we quantified the “accuracy of the positions” of the pixels with velocities higher than a given threshold. For this, we used the accuracy metric as defined in formula S.1.

$Accuracy= \frac{\left\| G\cap P \right\|}{\left\| G \right\|}$ (S.1)

where set $G=\{(i,j)|\left\| V_{g}(i,j) \right\|\geq g_{p}\}$ and set $P=\{(i,j)|\left\| V_{p}(i,j) \right\|\geq p_{p}\}$ contain the pixels whose resultant velocities $V$ are greater than a certain threshold. The threshold $g_{p}$ and $p_{p}$ are the *p*th percentile of the resultant velocity of ground truth and prediction, respectively.

## **S2. Results of input dimension**

**Table S.1**. Prediction results generated using different input dimensions and different velocity thresholds in 4CH and 2CH view. For EPE and angle error, >5 indicates that only the pixels in the ground truth with a velocity magnitude greater than 5cm/s are included to compute the metrics. ACC >30^th^ indicates that only the pixels with a velocity magnitude greater than 30^th^ percentile of all four chambers (LV,RV,LA, RA) in 4CH view and all two chambers (LV, LA) in 2CH view are included to compute the accuracy. *N* is the dimension of the network input. ACC means the evaluation metric accuracy. The best results within four different dimensions are shown in bold.

| View |  | EPE(cm/s) | | | Angle Error(°) | | | ACC(%) | | |
| --- | --- | --- | --- | --- | --- | --- | --- | --- | --- | --- |
|  |  | >0 | >5 | >10 | >0 | >5 | >10 | >30^th^ | >50^th^ | >70^th^ |
| 4CH | N=3 | 7.0±1.5 | 8.7±2.7 | 10.9±2.3 | 51.9±9.9 | 41.9±11.2 | 33.2±11.0 | 79.0±4.0 | 68.0±7.5 | 55.9±12.1 |
|  | N=5 | 7.0±1.5 | 8.7±2.6 | 10.8±2.3 | 51.7±10.0 | 41.6±11.3 | 32.9±11.2 | **79.2±3.9** | **68.4±7.5** | **56.4±11.9** |
|  | N=7 | **6.9±1.5** | **8.6±2.1** | 10.9±2.4 | 51.7±9.9 | 41.6±11.1 | 33.0±10.7 | 78.9±4.1 | 68.1±7.6 | 56.3±12.2 |
|  | N=9 | 6.9±1.5 | 8.6±2.7 | **10.8±2.4** | **51.4±10.1** | **41.3±11.4** | **32.7±11.0** | 79.1±4.0 | 68.2±7.7 | 56.3±12.1 |
| 2CH | N=3 | 7.2±1.8 | 9.1±2.0 | 11.8±2.6 | 56.9±10.9 | 47.2±11.9 | 36.5±12.4 | 78.8±4.9 | 66.7±9.5 | 53.5±16.0 |
|  | N=5 | 7.1±1.80 | 9.0±2.0 | 11.7±2.6 | 56.5±11.1 | 46.7±12.0 | 36.4±12.0 | 78.9±5.0 | 66.85±9.6 | 53.7±16.1 |
|  | N=7 | **7.1±1.8** | 9.0±2.0 | **11.6±2.5** | **56.4±11.1** | 46.6±12.0 | 36.4±12.4 | 78.8±5.0 | 66.9±9.6 | 53.8±16.2 |
|  | N=9 | 7.1±1.8 | **8.9±2.0** | 11.7±2.6 | 56.5±11.6 | **46.4±12.3** | **35.9±12.6** | **79.0±4.9** | **67.0±9.4** | **54.1±15.8** |

## **S3. Results in four-chamber view**

**Table S.2**. Prediction results of Accuracy in different chambers in 4CH view. Accuracy was computed using the 30^th^ percentile as the threshold. 4CH indicates the results were computed within all 4 chambers; LV, LA, RV and RA mean the results were based on each single chamber separately. The mean ± standard deviation are reported.

|  | 4CH | LV | LA | RV | RA |
| --- | --- | --- | --- | --- | --- |
| Accuracy (%) | 79.09±4.02 | 79.40±5.17 | 78.72±6.38 | 77.77±5.75 | 75.62±5.91 |

Intra-cardiac flow velocity varies greatly within the cardiac cycle, across regions, cardiac phases and also across patients. Hence, to further analyze the prediction results, various velocity thresholds were used to compute the evaluation metrics for those pixels exceeding a chosen threshold (as shown in Fig.S.1 and Fig.S.3). By excluding the low-velocity pixels, the performance of the model can be more clearly revealed.


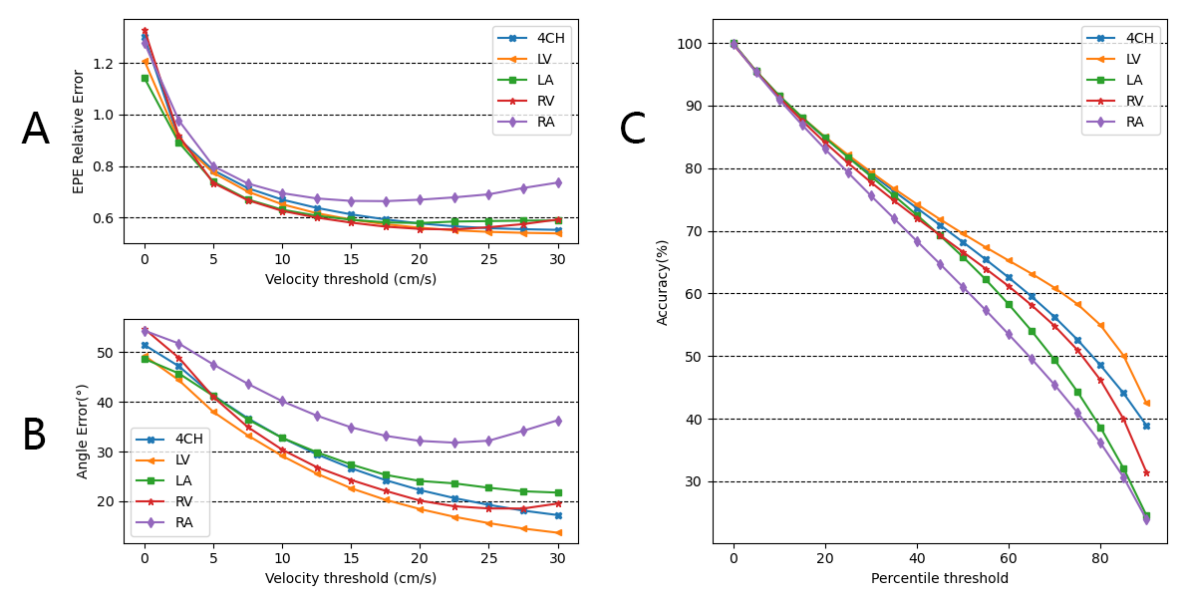


**Figure** S.1. Relative EPE, angle error and accuracy under different threshold values in different chambers in 4CH view. 4CH means all four chambers are included to compute the evaluation metrics. LV, LA, RV and RA means only one chamber was used to compute the metrics. (**A):** The relation between relative EPE, and the velocity threshold. (**B):** The relation between angle error and velocity threshold. (**C):** The relation between the accuracy and the velocity percentile threshold in different chambers.

It defined E/A ratio<0.5 as impaired relaxation pattern, 0.75< E/A ratio <1.5 as normal diastolic function and E/A ratio >2 as restrictive filling. The confusion matrix of the diastolic function classification experiment are summarized in Figure S.2. The diastolic function classification accuracy was 88.1%.


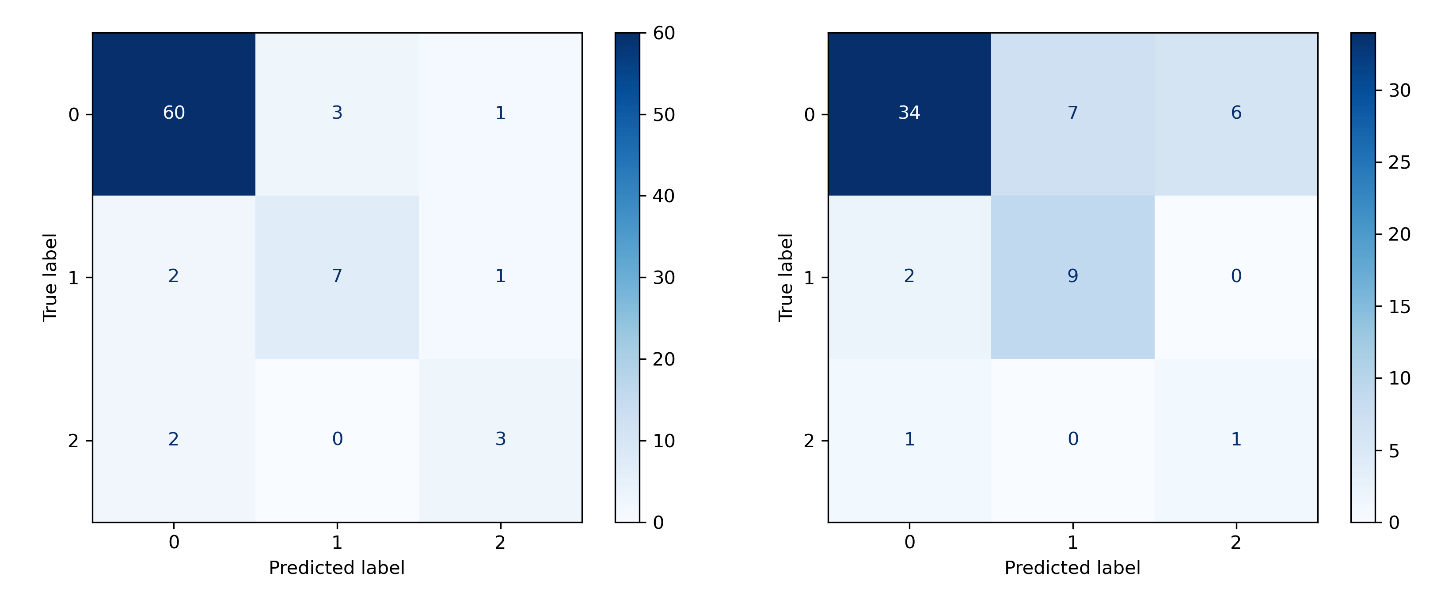


**Figure S.2.** Confusion matrix derived from the predicted velocities in the 4CH views. Label 0 means normal diastolic function, 1 is restrictive filling and 2 is impaired relaxation pattern.

We also test the performance of our model in two-chamber view. 86 cine 2CH views (2580 2D images) were used for training and testing.

## **S4. Results in two-chamber view**

**Table** S.3. Prediction results of different chambers in 2CH view. EPE and angle error were computed using a velocity threshold of 5 cm/s. Accuracy was computed using the 30^th^ percentile as the threshold. 2CH indicates the results were computed within all 2 chambers; LV, LA mean the results were based on each single chamber separately. PCC: Pearson correlation coefficient. The mean ± standard deviation are reported.

|  | 2CH | LV | LA |
| --- | --- | --- | --- |
| EPE (cm/s) | 8.99±2.02 | 9.18±2.15 | 8.78±2.40 |
| Angle Error (°) | 46.45±12.26 | 45.19±13.60 | 48.91±16.12 |
| Accuracy (%) | 79.03±4.93 | 79.90±5.72 | 76.21±6.76 |
| Velocity-RE (%) | -32.29±4.08 | -31.46±4.68 | -33.12±8.25 |
| Velocity-PCC | 0.971 | 0.984 | 0.869 |


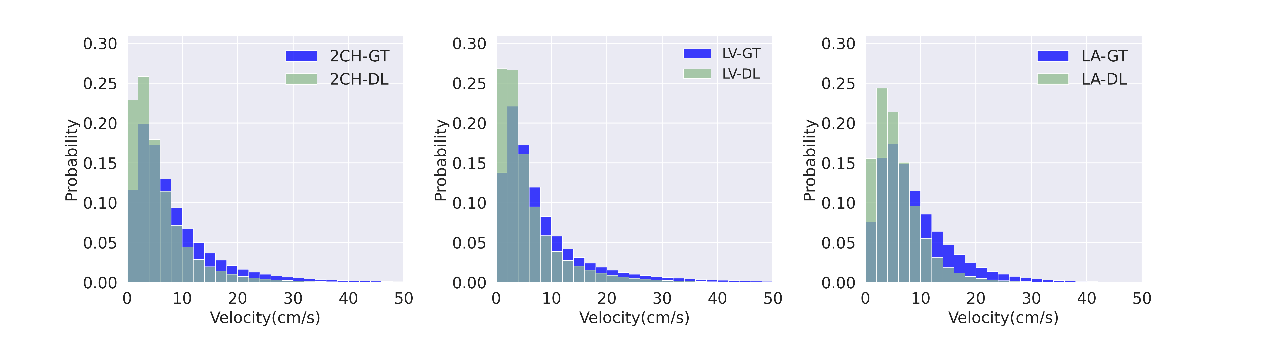


**Figure** S.3. Probability distribution of velocity generated from 4D flow data and prediction in 2CH view. The blue color represents the distribution generated from the 4D flow data, and the light green means the distribution generated from the prediction. The light blue represents the overlap between the prediction and 4D flow data.


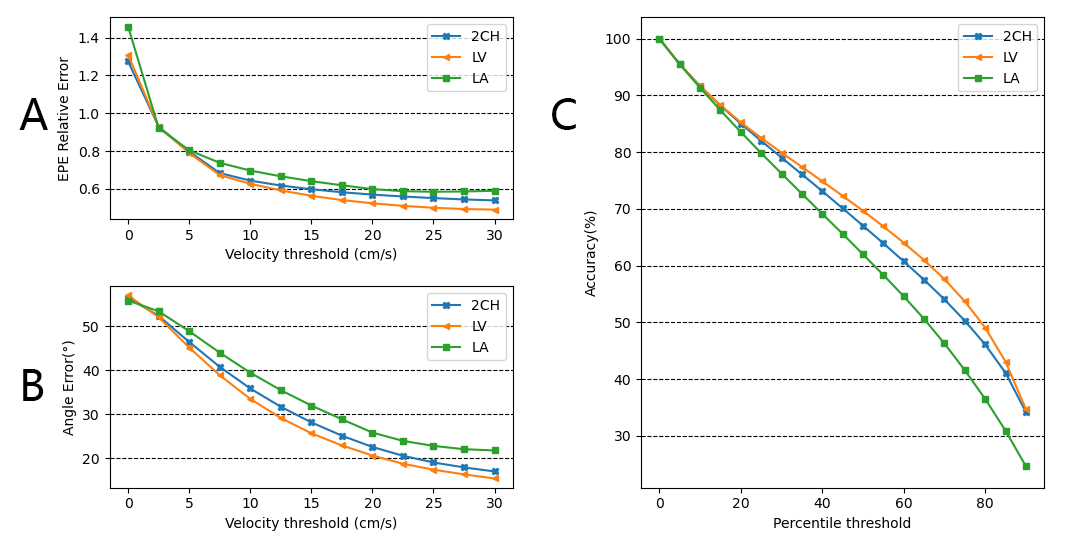


**Figure** S.4. Relative EPE, angle error and accuracy under various thresholds in different chambers in 2CH view. 2CH means LV and LA are included to compute the evaluation metrics. LV, LA means only one chamber was used to calculate the metrics. (**A):** The relation between relative EPE and velocity threshold. (**B):** The relation between angle error and velocity thresholds. (**C):** The relation between the accuracy and the velocity percentile threshold in different chambers.

The average absolute error in E/A ratio estimation in 2CH view was 0.46±0.42. In the 2CH view, there are 47 subjects with normal diastolic function, of those 47 subjects, seven were classified as having restrictive filling and six as having impaired relaxation. Two out of eleven subjects with restrictive filling were classified as normal diastolic function. The confusion matrix of the diastolic function classification experiment are summarized in Figure S.6. The classification accuracy in 2CH view was 73.3%. The Wilcoxon signed-rank test with P=.67 in 2CH view, confirmed that the E/A ratio generated from our prediction was not significantly different from the 4D flow data.


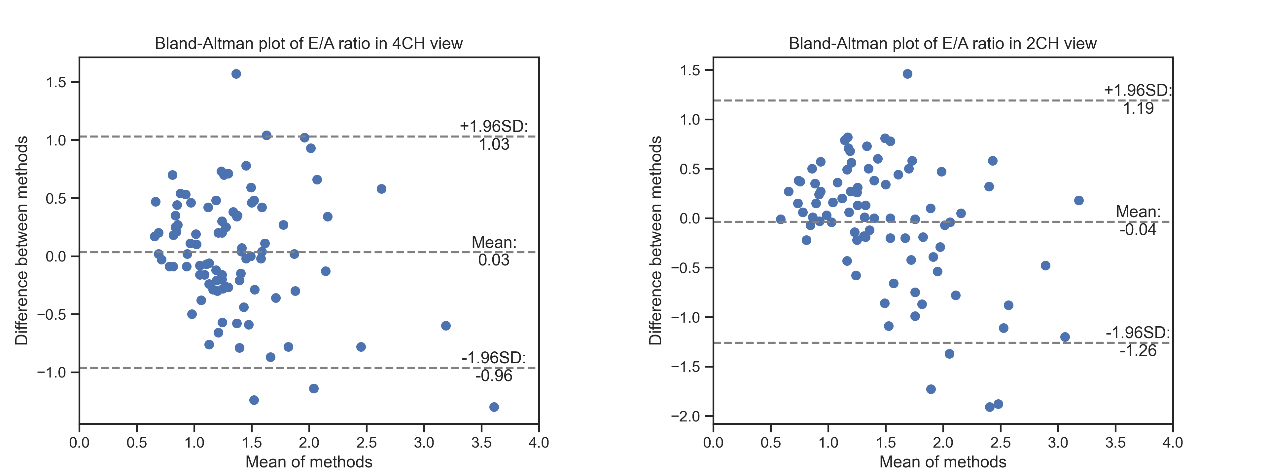


**Figure S.5.** Confusion matrix derived from the predicted velocities in the 4CH (left) and 2CH (right) views. Label 0 means normal diastolic function, 1 is restrictive filling and 2 is impaired relaxation pattern.


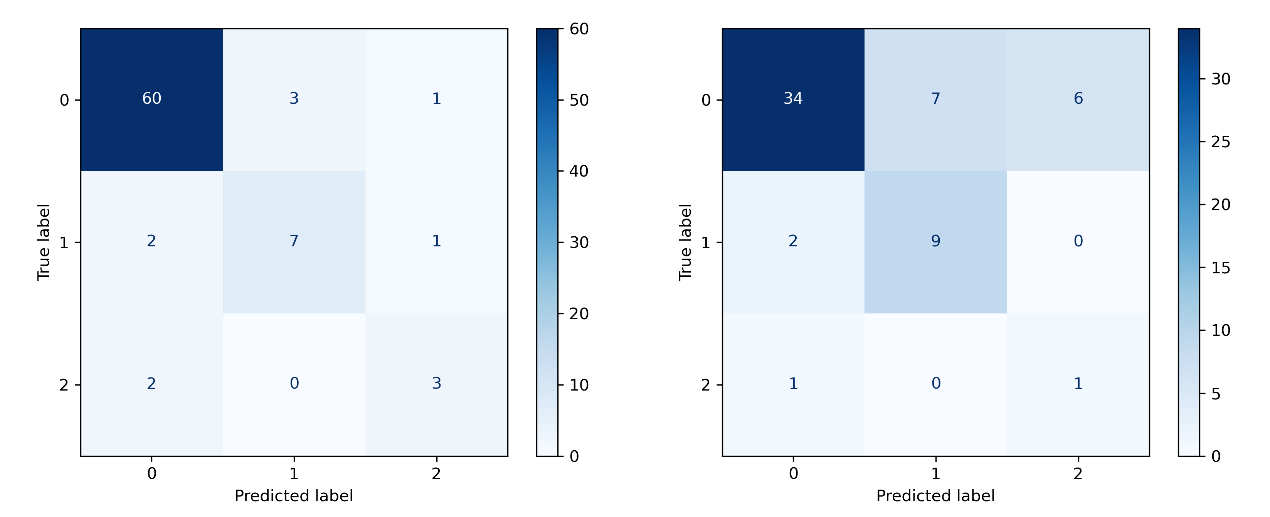


**Figure S.6.** Confusion matrix derived from the predicted velocities in the 2CH views. Label 0 means normal diastolic function, 1 is restrictive filling and 2 is impaired relaxation pattern.
